# Local salt substitutes “Obu-otoyo” activate acetylcholinesterase and butyrylcholinesterase and induce lipid peroxidation in rat brain

**DOI:** 10.1515/intox-2015-0021

**Published:** 2015-09

**Authors:** Ayodele J. Akinyemi, Ganiyu Oboh, Adedayo O. Ademiluyi

**Affiliations:** 1 Functional Foods and Nutraceuticals Unit, Department of Biochemistry, Federal University of Technology, Akure, P.M.B. 704, Akure, 340001, Nigeria; 2 Department of Biochemistry, Afe Babalola University, Ado-Ekiti, P.M.B. 5454, Ado-Ekiti, Nigeria

**Keywords:** Obu-Otoyo, lipid peroxidation, heavy metals, acetylcholinesterase, butyrylcholinesterase

## Abstract

Evidence has shown that ingestion of heavy metals can lead to neurodegenerative diseases. This study aimed to investigate the neurotoxic potential of salt substitutes (Obu-Otoyo); salt A (made by burning palm kernel shaft then soaked in water overnight and the extract from the resulting residue is used as the salt substitute) and salt B (an unrefined salt mined from a local site at Ilobu town, Osun-State, Nigeria) by assessing their effect on some key enzymes linked with neurodegenerative disease [acetylcholinesterase (AChE) and butyrylcholinesterase (BChE) activities] as well as on malondialdehyde (MDA) content of the rat brain. Salt substitutes were fed to normal rats as dietary inclusion at doses of 0.5 and 1.0% for 30 days. Thereafter, the effect of the salt substitutes on AChE and BChE activities as well as on MDA level in the rat brain was determined. The results revealed that the salt substitutes caused a significant (*p*<0.05) increase in both AChE and BChE activity and also induced lipid peroxidation in the brain of rats in vivo as well as under in vitro condition in a dose-dependent manner. The effect of the salt substitutes on AChE and BChE activities could be attributed to the presence of some toxic heavy metals. Therefore, the ability of the salt substitutes to induce lipid peroxidation and activate AChE and BChE activities could provide some possible mechanism for their neurotoxic effect.

## Introduction

Heavy metals have been linked to the formation of free radicals and could cause serious health problems with varied signs and symptoms depending on the nature and quantity of the metal ingested (Adepoju-Bello & Alabi, 2005). They produce their toxicity by depletion of glutathione and by forming complexes with proteins, in which carboxylic acid (–COOH), amine (–NH_2_), and thiol (–SH) groups are involved. Metal-induced formation of free radicals causes various modifications to DNA bases, enhanced lipid peroxidation, and altered calcium and sulfhydryl homeostasis (Valko *et al*., 2005). Generally, oxidative damage to cellular components results in alteration of the membrane properties such as fluidity, ion transport, enzyme activities, and protein cross-linking (Bandopadhyay *et al*., 1999). Oxidative damage, caused by the action of free radicals, may initiate and promote the progression of a number of chronic diseases, including cancer, cardiovascular diseases, inflammation, diabetes and Alzheimer's disease (Halliwell & Gutteridge, 1989).

Human exposure to heavy metals is a global public health problem (Neal & Guilarte, 2012). Heavy metals, such as lead (Pb^2+^) and manganese (Mn), which cause neurological toxicity, are of particular concern due to the long-lasting and possibly irreversible nature of their effects. Pb^2+^ exposure in childhood can result in cognitive and behavioral deficits in children (Bouchard *et al*., 2011). These effects are long-lasting and persist into adulthood even after reduction or elimination of Pb^2+^ exposure (Toscano & Guilarte, 2005). While Mn is an essential element of the human diet and serves many cellular functions in the human body, elevated Mn levels can result in a Parkinson disease (PD)-like syndrome and developmental Mn exposure can adversely affect childhood neurological development (Guilarte, 2010). Due to the ubiquitous presence of both metals and the fact that these metals can be absorbed in the body via food and drinking water, reduction of human exposure to toxic levels of Mn and Pb^2+^ remains a world-wide public health challenge.

Salt substitutes have been used as a replacement for common salt which is high in sodium content. High salt intake has long been suspected to raise blood pressure, which is attributed to increased plasma volume (Martel & Cassidy, 2004). A salt substitute does not taste exactly like sodium chloride but it is similar enough and contains less sodium. One important unrefined salt known as “Obu-Otoyo” from a local source in Nigeria has been reported to be used as salt substitute, applied also in the diet of patients for the management of diabetes and hypertension. In Nigeria, two salt substitutes, which differ in color and origin, are popular among the local people; salt substitute “A”, grey in color, is the ash from burnt palm kernel shaft that was soaked in water overnight and the residue concentrated to produce the salt substitute, while salt substitute “B”, white in color, is a rock salt mined from a local site at Ilobu town, Osun-State, Nigeria. These salt substitutes are generally used for cooking and food preservation, as well as for other purposes such as production of soaps and other bath products, and as detergent (Kilani *et al*., 2007). Hence the present study sought to investigate the effect of the salt substitutes (Obu-Otoyo), salt A and salt B, on some key enzymes linked with neurodegenerative disease [acetylcholinesterase (AChE) and butyrylcholinesterase (BChE) activities] as well as on the malondialdehyde (MDA) content of rat brain *in vitro* in order to provide some evidence for the neurotoxic effect of these salt substitutes.

## Materials and methods

### Sample collection

Salt substitute (A and B) samples were obtained from the Oja Oba market in Akure metropolis from two different sources. The two salts are of different color and origin. Salt substitute “A”, grey in color, is the ash from burnt palm kernel shaft that was soaked in water overnight and the residue concentrated to produce the salt substitute, while salt substitute “B”, white in color, is a rock salt mined from a local site at Ilobu town, Osun-State, Nigeria.

### Chemicals and reagents

Chemicals and reagents used such as malondialdehyde tetrabutyl ammonium salt (standard MDA), thiobarbituric acid (TBA), trichloroacetic acid, acetic acid and 5,5´-dithio-bis(2-nitrobenzoic) acid (DTNB) were sourced from Sigma-Aldrich, Chemie GmbH (Steinheim, Germany), acetylthiocholine iodide and s-butyrylthiocholine iodide were procured from Sigma-Aldrich, Inc., (St. Louis, MO, USA). All other chemicals used were of analytical grade, while the water was glass distilled.

### Experimental animals

Wistar strain male Albino rats weighing 100–150 g from the Department of Biochemistry, Federal University of Technology, Akure, were fed commercially available standard pelleted diet and water *ad libitum*. The handling and use of the animals were in accordance with the NIH Guide for the care and use of laboratory animals.

### Bioassay

The bioassay was carried out according to the method described by Oboh *et al*. (2012). Nigeria. The study was approved by the Animal Ethics Committee of the Federal University of Technology, Akure, Nigeria. After acclimatization for two weeks, the rats were divided into five groups. Group 1 served as control and was placed on a basal diet [skimmed milk (44.4%), corn starch (41.6%), groundnut oil (10%) and vitamin-mineral premix (4%)]. Groups 2 and 3 were fed basal diet plus 0.5% and 1.0% inclusion of salt substitute A, respectively, while groups 4 and 5 were fed basal diet plus respective 0.5% and 1.0% inclusion of salt substitute B. Daily food intake was monitored and body weight was taken both at the beginning and end of the experiment. The experiment lasted for 30 days after which the rats were sacrificed by cervical dislocation and their brains were immediately removed, rinsed in ice-cold 1.15% KCl, blotted and weighed. The brains were then minced with scissors in three volumes of ice-cold 100 mM potassium phosphate buffer, pH 7.4 and homogenized in a teflon glass homogenizer. The homogenates were centrifuged for 10 min at 12,000 × *g* to yield a pellet that was discarded, and a lowspeed supernatant (S1) which was used to assess the activities of acetylcholinesterase (AChE) and butyrylcholinesterase (BChE) (Ellman *et al*., 1961), as well as to determine the malondialdehyde (MDA) level (Ohkawa *et al*., 1979) and protein content (Gornall *et al*., 1949).

### *In vitro* analysis

#### Mineral determination

An appropriate hollow cathode lamp was fixed according to the element that was to be determined. An atomic absorption spectrophotometer was set and allowed to stabilize using standard solutions. The stock standard for each mineral was 10 mg/kg. The standard for each metal was aspirated into the flame and the absorbance of the standards was noted.

#### Acetylcholinesterase (AChE) and butyrylcholinesterase (BChE) activities

The effect of the salt substitutes on acetylcholinesterase (AChE) and butyrylcholinesterase (BChE) activities was assessed by using the colorimetric method of Ellman *et al*. (1961). The AChE activity was determined in a reaction mixture containing 200 μL of brain AChE solution in 0.1 M phosphate buffer, pH 8.0, 100 μL of a solution of 5,5´-dithio-bis(2-nitrobenzoic) acid (DTNB 3.3 mM in 0.1 M phosphate buffered solution, pH 7.0, containing NaHCO_3_ 6 mM), salt sample (0–576.92 μg/mL) and 500 μL of phosphate buffer, pH 8.0. After incubation for 20 minutes at 25 °C, acetylthiocholine iodide (100 μL of 0.05 mM water solution) was added as substrate and AChE activity was determined by UV spectrophotometry from the absorbance changes at 412 nm for 3.0 minutes at 25 °C. Hundred microliters of butyrylthiocholine iodide was used as a substrate to assay butyrylcholinesterase enzyme, while all the other reagents and conditions were the same. The AChE and BChE activity was expressed as percentage of relative activity.

#### Fenton reaction (degradation of deoxyribose)

The ability of the salt substitutes to cause salt/H_2_O_2_ induced decomposition of deoxyribose were carried out using the modified method of Halliwell and Gutteridge (1981). The salt substitutes, 0–86.9 μg/mL, were added to the reaction mixture containing 120 μL of 20 mM deoxyribose, 400 μL of phosphate buffer, 40 μL of H_2_O_2_ and the volume was made up to 800 μL with distilled water. The reaction mixture was incubated at 37 °C for 30 minutes. Then 500 μL of trichloroacetic acid was added followed by the addition of 400 μL of 0.6% thiobarbituric acid solution. The tubes were subsequently incubated in boiling water for 20 minutes. The absorbance was measured at 532 nm in a spectrophotometer.

### Lipid peroxidation assay

#### Preparation of tissue homogenates

The rats were decapitated under mild diethyl ether anesthesia and the tissues (brain) were rapidly dissected and placed on ice and weighed. This tissue was subsequently homogenized in cold saline (1/10 w/v) with about 10 up-and-down strokes at approximately 1200 rev/min in a Teflon glass homogenizer (Mexxcare, mc14 362, Aayushi Design Pvt. Ltd. India). The homogenate was centrifuged (KX3400C Kenxin Intl. Co. Hong Kong) for 10 minutes at 3,000 × g to yield a pellet, which was discarded, and a low-speed supernatant (SI) which was kept for lipid peroxidation assay (Belle *et al*., 2004).

#### Lipid peroxidation and thiobarbibutric acid reactions

The lipid peroxidation assay was carried out using the method of Ohkawa *et al*. (1979), briefly 100 μL of the SI fraction was mixed with a reaction mixture containing 30 μL of 0.1 M pH 7.4 Tris-HCl buffer and salt substitutes (0–75 μg/mL). The volume was made up to 300 μL by water before incubation at 37 °C for 2 hours. The color reaction was developed by adding 300 μL 8.1% SDS (sodium dodecyl sulphate) to the reaction mixture containing SI. This was subsequently followed by the addition of 600 μL of acetic acid/HCl (pH 3.4) mixture and 600 μL of 0.8% thiobarbituric acid (TBA). This mixture was incubated at 100 °C for 1 hour. Thiobarbituric acid reactive species (TBARS) produced were measured at 532 nm and expressed as malondialdehyde (MDA) produced (% control) using the malondialdehyde standard curve (0–0.035 mM).

### Data analysis

The results of three replicate experiments were pooled and expressed as mean ± standard deviation (STD). One-way analysis of variance and the least significance difference were carried out using SPSS software package for Windows (SPSS Inc., Chicago, IL, USA). Significance was accepted at *p*<0.05 (Zar *et al*., 1984).

## Results

The result of mineral composition analysis of the salt substitute “Obu-Otoyo” is shown in Table 1 and is expressed as mg/kg. The result revealed that both salt substitutes (A and B) contained high amounts of macro-elements (K, Na, Ca and Mg) and micro-elements (Cu, Co, Cd, Pb, Ni, Fe, Cr, Mn and Zn).

**Table 1:** Mineral composition of salt substitutes “Obu-Otoyo” in mg/kg.

Minerals	Salt Substitute A	Salt Substitute B
K	237332.5^a^±10.5	1649.3^b^±1.4
Na	6632.1^a^±1.3	383263.3^b^±9.6
Ca	4270.5^a^±1.70	2523.7^b^±2.7
Mg	534.1^a^±1.4	151.8^b^±1.0
Cu	5.7^a^±0.1	3.7^b^±0.1
Co	21.0^a^±1.1	20.5^a^±1.0
Cd	1.7^a^±0.1	3.7^b^±0.1
Pb	16.7^a^±1.8	27.7^b^±1.9
Ni	7.4^a^±0.8	19.63^b^±1.0
Fe	234.8^a^±3.8	119.7^b^±2.7
Cr	3.2^a^±0.8	3.6^a^±0.5
Mn	7.5^a^±0.8	6.4^a^±0.8
Zn	21.6^a^±1.8	2.8^b^±0.1

Values represent mean ± standard deviation, number of samples n = 6. Values with the same superscript letter along the same row are not significantly different (*p*>0.05).

**Figure 1:**
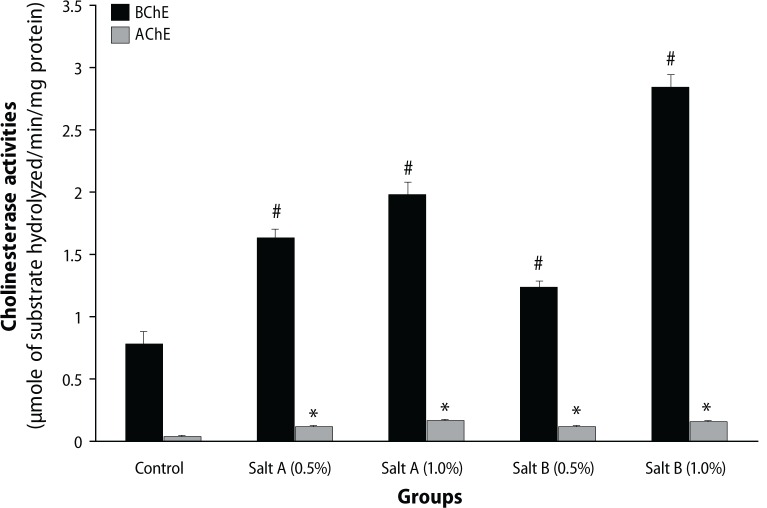
Effect of dietary inclusion (0.5 and 1.0%) of salt substitute “Obu-Otoyo” on cholinesterase activities in rats (*n*=6). *# Significantly different from control group at *p*<0.05. BChE – Butyrylcholinesterase; AChE – Acetylcholinesterase.

**Figure 2:**
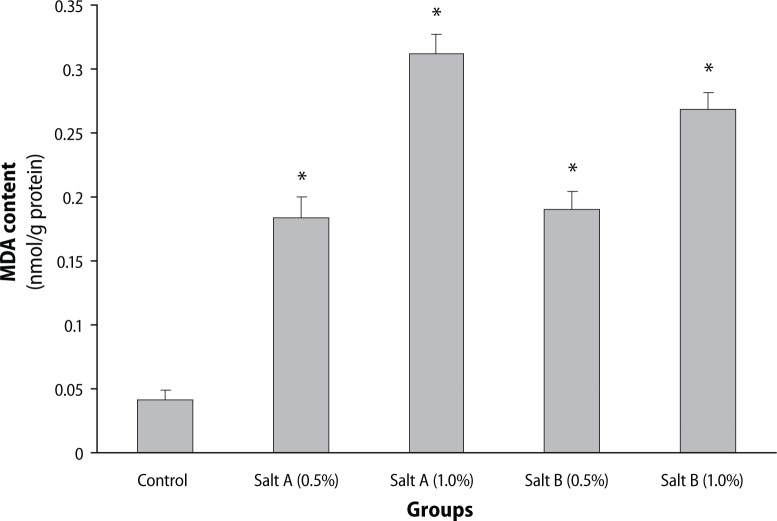
Effect of dietary inclusion (0.5 and 1.0%) of salt substitute “Obu-Otoyo” on brain malondialdehye (MDA) content in rats ^*^Significantly different from control group at *p*<0.05.

As presented in Figure 1, dietary inclusion of the salt substitute “Obu-Otoyo” to diets caused a significant (*p*<0.05) increase of AChE and BChE activities when compared with control. There was also an increase in MDA level by dietary inclusion of the salt substitute “Obu-Otoyo” to diets compared with control (Figure 2).

**Figure 3:**
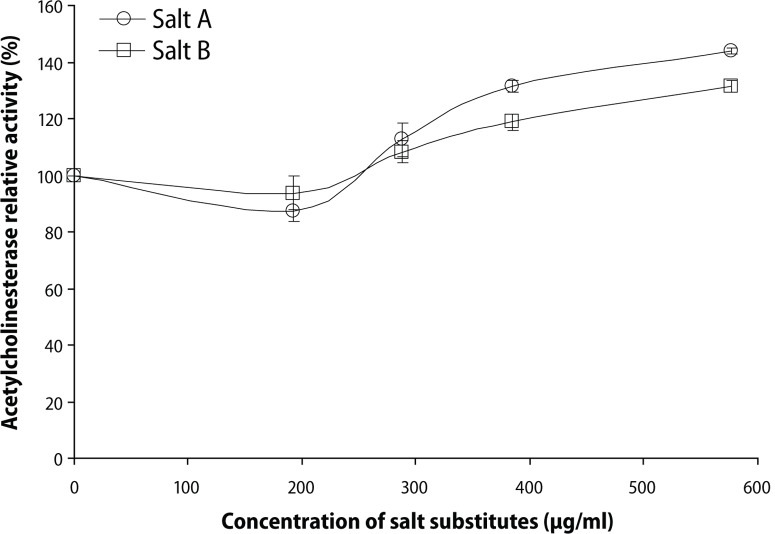
Effect of salt substitute “Obu-Otoyo” on acetylcholinesterase (AChE) activity in vitro.

**Figure 4:**
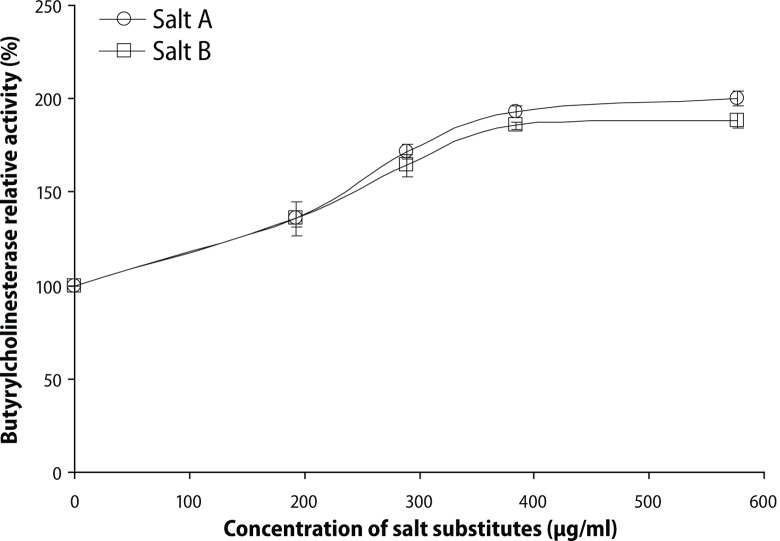
Effect of salt substitute “Obu-Otoyo” on butyrylcholinesterase (BChE) activity *in vitro*.

Furthermore, the ability of the salt substitutes to induce acetylcholinesterase (AChE) and butyrylcholinesterase (BChE) activities under in *vitro* conditions was investigated and the result is presented in the respective Figures 3 and 4. The result revealed that the interaction of the salt substitutes with AChE and BChE caused a significant (*p*<0.05) increase in the enzyme activities. Yet there was no significant difference (*p*>0.05) in the AChE and BChE activity induced by the two salt substitutes.

**Figure 5:**
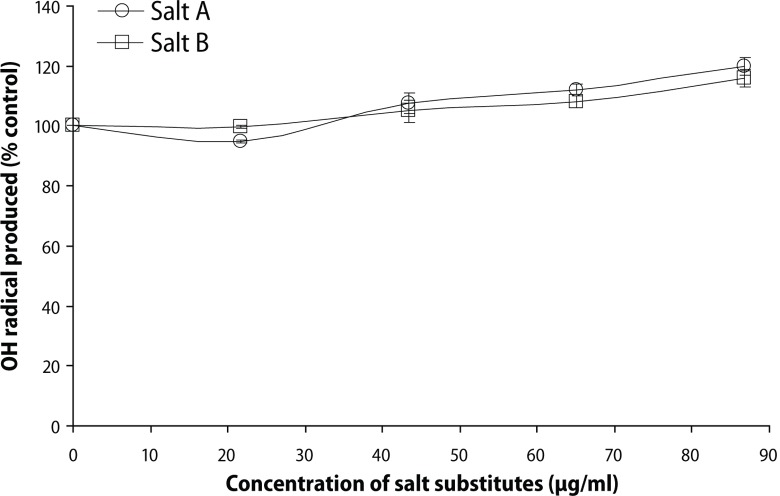
Salt substitute “Obu-Otoyo” ability to enhance OH radical formation in Fenton reaction.

The ability of the salt substitute “Obu-Otoyo” to enhance the production of hydroxyl radical in Fenton reaction is presented in Figure 5. The result revealed that the salt substitutes “Obu-Otoyo” A and B induced the formation of hydroxyl radical (OH^•^) from the decomposition of deoxyribose in Fenton reaction in a dose-dependent manner (21.7–86.9 μg/mL).

**Figure 6:**
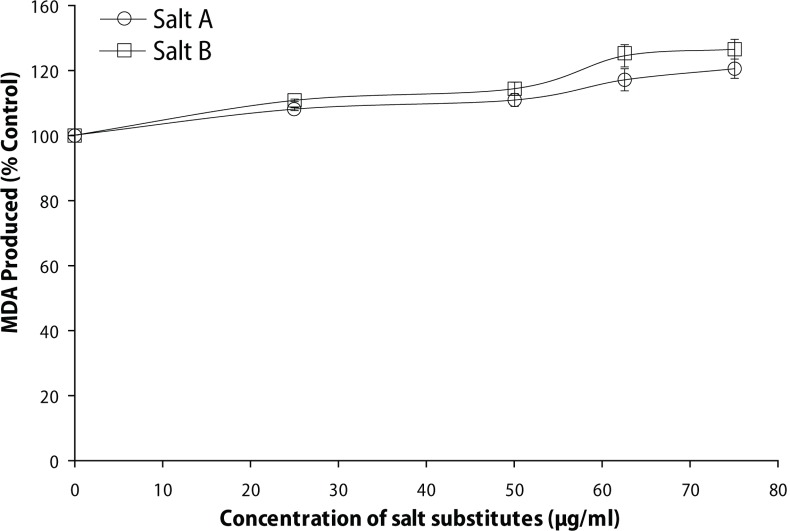
Effect of salt substitute “Obu-Otoyo” on malondialdehyde (MDA) content of rat brain.

Furthermore, the ability of the salt substitute “Obu-Otoyo” to induce lipid peroxidation in the rat brain *in vitro* was also investigated and the result is presented in Figure 6. The salt substitutes were found to induce lipid peroxidation *in vitro* as revealed by the increase in malondialdehyde (MDA) produced in the brain in a dose-dependent manner (25–75 μg/mL). However, there was no significant (*p*<0.05) difference between the two salt substitutes in inducing lipid peroxidation in rat brain.

## Discussion

Heavy metal pollution is a global public health challenge, with a disproportionate burden laid upon developing nations. The developing world, with increased heavy metal contamination and higher prevalence of dietary deficiencies, is at particular risk for metal toxicity. Generally, humans are not exposed to a single heavy metal but rather to heterogeneous metal mixtures. The effect of human exposure to mixtures of heavy metals is currently an active area of research.

The use of Obu-Otoyo as salt substitute has been a usual practice in traditional folklore for several centuries, employed also in the diets of patients suffering from hypertension. Our results revealed that the salt substitutes “Obu-Otoyo” contained some toxic/heavy metals such as Co, Cd, Pb, Cr and Zn (Table 1). These heavy metals could cause serious health problems with varied signs and symptoms, depending on the nature and quantity of the metal ingested (Valko *et al*., 2005).

Heavy metals such as lead (Pb^2+^) and manganese (Mn) have been shown to induce cognitive and behavioral deficits in adults and children at elevated levels of exposure (Guilarte, 2010; Toscano & Guilarte, 2005). They can result in distinct neurological effects, with different brain targets, and their mode of action is probably by disrupting presynaptic neurotransmission.

Absorption of heavy metals from the intestine is mediated by both passive and facilitated diffusion, although passive diffusion plays a minor role in total absorption (Aungst & Fung, 1981). Studies on the intestinal absorption of Pb^2+^ have consistently reported evidence of carrier-mediated transport (Barton *et al*., 1978; Fullmer, 1990). Some evidence supports the hypothesis that the divalent metal transporter 1 (DMT1) is responsible for transporting heavy metals like lead (Pb^2+^), zinc (Zn^2+^) and cadmium (Cd^2+^) (Bannon *et al*., 2003). DMT1 is a metal ion transporter that can transport heavy metals in addition to its physiological substrate, iron (Fe) (Bannon *et al*., 2003). Thus, while DMT1 is likely to play a role in metal uptake from the gastrointestinal tract, it is apparent that other carrier proteins do exist. In blood, heavy metals are primarily bound to protein. Up to 40% of blood metals bind to serum albumin and the remaining to sulfhydryl- or thiol-containing ligands (Al-Modhefer *et al*., 1991). Work with the radiotracer 203-Pb in rats has demonstrated that Pb^2+^ is taken up into the brain most likely as a free ion (PbOH^+^) or complexed with small molecular weight ligands. PbOH+ crosses presumably the blood brain barrier (BBB) through passive diffusion, but could also be transported through cation transporters (Yokel, 2006). DMT1 is highly expressed in the striatum, cortex, hippocampus, and cerebellum (Williams *et al*., 2000) and may facilitate heavy metal transfer across the BBB (Wang *et al*., 2011). Brain efflux is likely to be mediated through ATP-dependent Ca^2+^ pumps (Marchetti, 2003).

In our study, dietary inclusion of the salt substitute “Obu-Otoyo” at 0.5 and 1.0% in rat diets caused a significant (*p*<0.05) increase in both acetylcholinesterase (AChE) and butyrylcholinesterase (BChE) activities in the brain when compared with control rats. The increase in both AChE and BChE could be a result of some of the toxic heavy metals present in the salt substitutes (Table 1). Recent reports have indicated that some metals such as lead, copper and iron can activate AChE activity during acute exposure (Bainy *et al*., 2006). Based on these results, we can suggest that exposure of rats to these salt substitutes may interact with acetylcholine/butyrylcholine receptors through the metals, thereby affecting the binding efficiency, leading to an increase in AChE/BChE activity, and decomposition of the higher levels of the neurotransmitter.

Acetylcholine, the principal neurotransmitter of the cholinergic neurons, is one of the main neurotransmitters involved in neurodegenerative diseases (Wacker *et al*., 2005) and is related to cognitive functions involved in learning and memory processes (Blockland, 1995). Cholinergic neurons correspond to 25% of the brain cells and are represented mainly by cortical and hippocampal neurons (Wacker *et al*. 2005). The synaptic cholinergic transmission depends on acetylcholinesterase (AChE) (E.C.3.1.1.7) activity since this enzyme promotes the hydrolysis of the neurotransmitter acetylcholine in choline and acetic acid, resulting in the terminus of the transmission of the nervous impulse in the synapses (Taylor, 1996). This result is in agreement with Gill *et al*. (1991), where an increased AChE activity in skeletal muscles and brain of the fish *Barbus conchonius* was observed when exposed to cadmium and lead. Romani *et al*. (2003) have also suggested that metals under laboratory conditions interact with AChE receptors, thereby affecting its binding efficiency, which leads to an increase in AChE activity. Gallegos *et al*. (2001) observed after 30 minutes an increase of AChE activity in the brain of rats exposed to 10 mg/kg of Pb. One possible mechanism by which Pb^2+^ causes neurotoxicity is that it targets the learning and memory processes of the brain by inhibiting the N-methyl-D-aspartate receptor (NMDAR), which is essential for hippocampus-mediated learning and memory (Morris *et al*., 1986). The NMDAR is essential for learning spatial navigation tasks in animal models (Morris *et al*., 1986) and animals which have been developmentally exposed to Pb^2+^ exhibit similar learning deficits as those with absent or impaired NMDARs (Morris *et al*., 1986; Tsien *et al*., 1996).

Furthermore, we also studied the *in vitro* effects of the interaction of the salt substitutes with AChE and BChE activity of the brain of rats of the same age as used in the *in vivo* study. The result revealed that the interaction of the salt substitutes with AChE and BChE caused significant (*p*<0.05) increase in the enzyme activities in a dose dependent manner (0–576.9 μg/mL). Nevertheless, there was no significant difference (*p*>0.05) in the AChE and BChE activity of the two salt substitutes. This result is in agreement with our earlier result in Figure 1, where dietary inclusion of the salt substitute “Obu-Otoyo” at 0.5 and 1.0% in rat diets caused a significant (*p*<0.05) increase in both AChE and BChE activity. The enhanced activity of AChE and BChE is detrimental to patients suffering from Alzheimer's disease (Orhan *et al*., 2004). Consumption of these salt substitutes is very harmful to health as it could initiate neurodegeneration by enhancing AChE and BChE activities.

In addition, the present study resulted in increased lipid peroxidation as evident by the significant elevation in brain MDA level (Figure 2). Reports have indicated that heavy metals act as catalysts in oxidative reactions of biological macromolecules and thus the toxicities of these metals might be due to oxidative tissue damage (Stohs & Bagchi, 1995). Redox-active metals, such as Fe, Cu and Cr, undergo redox cycling whereas redox-inactive metals, such as Pb, Cd, Hg and others, deplete the cells’ major antioxidants, particularly thiol-containing antioxidants and enzymes (Valko *et al*., 2005). Both redox-active or redox-inactive metals may cause an increase in the production of ROS such as hydroxyl radical (^•^OH), superoxide radical (O_2_^•-^) or hydrogen peroxide (H_2_O_2_), reacting with polyunsaturated fatty acids forming lipid peroxides that may in turn generate malondialdehyde (MDA). This is also evident by the significant increase in MDA production in the rat brain *in vitro* (Figure 6). The ability of the salt substitute to induce lipid peroxidation *in vitro* could be attributed to the presence of toxic heavy metals such as Pb, Cr, Cd, Fe and Cu. Earlier findings have shown that Fe^2+^ caused a significant increase in the MDA content of the rat brain *in vitro* (Oboh *et al*., 2007). Fe^2+^ and Cu^2+^ have been shown to be potent initiators of lipid peroxidation in the brain (pro-oxidant). Fe^2+^ can catalyze oneelectron transfer reactions that generate reactive oxygen species, such as the reactive OH·, which is formed from H_2_O_2_ through the Fenton reaction (Oboh *et al*., 2007). Iron also decomposes lipid peroxides, thus generating peroxyl and alkoxyl radicals, favoring the propagation of lipid oxidation (Zago *et al*., 2000). Elevated Fe^2+^ content in the brain has been linked to a host of neurodegenerative diseases. Elevated Fe levels have been localized to degenerate regions of brains from Alzheimer's disease patients, a finding also demonstrated in animal models of the disease. The mechanism of action by which the salt substitutes induce lipid peroxidation in the rat brain is probably due to the fact that they could induce the formation of hydroxyl radical (OH·) from the decomposition of deoxyribose in Fenton reaction in a dose-dependent manner (Figure 5). The attack of radicals on polyunsaturated fatty acid residues of phospholipids results in the production of lipid peroxides which could react with redox active metals to produce malondialdehyde, 4-hydroxynonenal and other exocyclic DNA adducts (Valko *et al*., 2005).

## Conclusion

In conclusion, local salt substitutes induce lipid peroxidation and enhance the activities of key enzymes linked to neurodegenerative diseases, which could be caused by the presence of some toxic heavy metals such as Pb, Cd and Cr. Thus the salt substitutes “Obu-Otoyo” possess some neurotoxic potential through their ability to enhance the activity of AChE and BChE, which makes them detrimental to the health of people consuming them.
